# Euglycemic Ketoacidosis in a Non-diabetic Patient After Tirzepatide Use: A Cautionary Tale

**DOI:** 10.7759/cureus.83187

**Published:** 2025-04-29

**Authors:** Renisha Singh

**Affiliations:** 1 Gastroenterology, The Hillingdon Hospital, Uxbridge, GBR

**Keywords:** adverse drug reaction, euglycemic ketoacidosis, glp-1 agonist, medical weight loss, mounjaro, obesity, obesity pharmacotherapy, tirzapetide

## Abstract

Tirzepatide, a dual glucagon-like peptide-1 (GLP-1) and glucose-dependent insulinotropic polypeptide (GIP) receptor agonist, has emerged as a promising pharmacological agent for weight loss in both diabetic and non-diabetic individuals. However, its widespread use, particularly without close medical supervision, raises concerns about rare but serious adverse effects.

We describe a case of a 48-year-old woman with a BMI of 30.4 kg/m² who initiated tirzepatide therapy via an online prescription. She self-titrated to a weekly dose of 5 mg over six weeks, achieving a 16-pound weight loss. One week prior to admission, she developed persistent diarrhea, appetite loss, nausea, and abdominal pain. On presentation, she was clinically dehydrated, with laboratory findings revealing a pH of 7.2, bicarbonate level of 17 mmol/L, and elevated ketones, while serum glucose remained normal. A diagnosis of tirzepatide-induced euglycemic ketoacidosis (EKA) was made. She was managed conservatively with fluid resuscitation and recovered without complications.

This case highlights EKA as a rare but potentially serious adverse effect of tirzepatide, particularly in the context of appetite suppression and caloric restriction. It emphasizes the need for medical supervision and patient education when prescribing novel weight-loss agents.

## Introduction

Obesity remains a pressing global health issue, with the World Health Organization (WHO) reporting that in 2023, over one billion adults worldwide were classified as obese [1]. In the UK, recent statistics show that approximately 27% of adults are living with obesity [2]. Obesity significantly increases the risk for a myriad of comorbid conditions, including type 2 diabetes mellitus, cardiovascular disease, cerebrovascular events, and several cancers.

Although lifestyle interventions such as diet and exercise are the cornerstone of management, pharmacological therapies have gained momentum for individuals who struggle to achieve meaningful weight loss through non-pharmacologic measures alone [3]. Among these, glucagon-like peptide-1 (GLP-1) receptor agonists, and more recently, dual GLP-1/glucose-dependent insulinotropic polypeptide (GIP) receptor agonists have emerged as powerful tools in the armamentarium against obesity.

Tirzepatide (Mounjaro™), a once-weekly injectable dual GLP-1/GIP receptor agonist, has shown significant promise in clinical trials such as SURMOUNT-1, demonstrating an average weight reduction of up to 22.5% at higher doses in non-diabetic patients [4]. Initiation typically starts at 2.5 mg once weekly, with dose increments of 2.5 mg every four weeks until a target dose (up to 15 mg weekly) is achieved based on tolerability and clinical response [5].

While the commonly reported side effects include nausea, vomiting, diarrhea, constipation, and abdominal discomfort [6], serious adverse events such as pancreatitis and gallbladder disease have also been documented. However, euglycemic ketoacidosis (EKA), particularly in non-diabetic individuals, remains exceedingly rare and underrecognized.

Herein, we present a rare and diagnostically challenging case of starvation ketoacidosis following unsupervised online prescription and use of tirzepatide.

## Case presentation

A 48-year-old woman with a body mass index (BMI) of 30.4 kg/m² and no significant past medical history sought an online consultation for weight loss. She worked a sedentary job in administration and had no history of diabetes, alcohol use, or eating disorders.

Following a virtual consultation, she was prescribed tirzepatide and initiated therapy at 2.5 mg weekly. Per standard practice, the recommended titration schedule advises dose escalation by 2.5 mg every four weeks to improve gastrointestinal tolerability [5]. In her case, she self-increased the dose to 5 mg after four weeks without any clinician follow-up. Over the next month, she reported a weight loss of 7.2 kg (16 pounds).

Approximately one week prior to admission, she developed persistent watery diarrhea, marked anorexia, generalized abdominal discomfort, and progressive fatigue. She denied vomiting but reported minimal oral intake. On arrival to the Emergency Department, she was hypotensive and clinically dehydrated.

Laboratory results revealed capillary ketones of 5.4 mmol/L (normal < 0.6 mmol/L), arterial blood gas of pH 7.27, pCO₂ 27 mmHg, bicarbonate 17 mmol/L, anion gap of 18 mmol/L (elevated), random glucose of 5.6 mmol/L (normoglycemia), and HbA1c of 6.0% (prediabetic range) (Table 1).

**Table 1:** Key laboratory parameters

Parameter	Patient Value	Reference Range
Capillary Ketones	5.4 mmol/L	0.0 – 0.6 mmol/L
Venous pH (initial)	7.27	7.35 – 7.45
Bicarbonate (initial)	17 mmol/L	22 – 29 mmol/L
Base Excess (initial)	-8	-2 to +2
pCO₂ (initial)	4.1 kPa	4.7 – 6.0 kPa
Anion Gap	18 mmol/L	8 – 16 mmol/L
Urea	9.8 mmol/L	2.5 – 7.8 mmol/L
Creatinine	122 µmol/L	45 – 90 µmol/L (female)
CRP	3 mg/L	<5 mg/L
Glucose	5.8 mmol/L	4.0 – 7.8 mmol/L (random
HbA1C	6 (pre-diabetic)	Equal to or less than 5.6

There were no signs of infection, sepsis, or alcohol-related illness. Imaging ruled out pancreatitis. Based on the clinical and biochemical profile, a diagnosis of euglycemic ketoacidosis was made, likely precipitated by reduced caloric intake in the setting of ongoing GLP-1/GIP receptor agonist use.

The patient was managed with aggressive intravenous fluid resuscitation, correction of electrolyte imbalances, and gradual reintroduction of oral nutrition. Serial biochemical assessments showed normalization of serum bicarbonate levels and ketones prior to discharge, confirming biochemical resolution. She showed clinical improvement within 72 hours and was discharged with strict advice to discontinue tirzepatide and seek weight management advice under medical supervision.

## Discussion

Tirzepatide exerts its weight loss effect through enhanced insulin secretion, delayed gastric emptying, and potent appetite suppression. While gastrointestinal side effects are well-characterized, cases of severe metabolic disturbances such as ketoacidosis remain rare.

EKA typically arises when insulin levels are relatively low, but not enough to cause hyperglycemia, resulting in unchecked lipolysis and ketogenesis [7]. In this case, the suppression of appetite led to a starvation state, reduced insulin action, elevated glucagon levels, and subsequent ketoacidosis despite normal blood glucose.

This diagnostic challenge is compounded by the absence of hyperglycemia, often leading to delays in recognition and treatment. Previous case reports, including the one by Wadden et al. [8], have described similar presentations, emphasizing the role of appetite suppression-induced caloric deprivation as a trigger for metabolic decompensation.

Compared to typical diabetic ketoacidosis, EKA in non-diabetic patients poses a unique diagnostic hurdle. Clinicians must maintain a high index of suspicion in any patient presenting with metabolic acidosis, elevated ketones, and normoglycemia, especially in the context of GLP-1 or dual agonist therapy.

In this case, unsupervised dose escalation, lack of monitoring of metabolic parameters, and inadequate patient education regarding the warning signs contributed to a near-critical outcome.

## Conclusions

Pharmacological weight loss therapies like tirzepatide offer substantial benefits but are not without risk. This case highlights a rare but serious adverse event associated with unsupervised use, underscoring the critical need for clinician involvement, patient counseling, and structured follow-up. As obesity pharmacotherapy continues to evolve, ensuring safe prescribing practices must remain a top priority.
